# Linguistic embodiment and verbal constraints: human cognition and the scales of time

**DOI:** 10.3389/fpsyg.2014.01085

**Published:** 2014-10-02

**Authors:** Stephen J. Cowley

**Affiliations:** Language and Communication, Centre for Human Interactivity and the COMAC Cluster, University of Southern DenmarkSlagelse, Denmark

**Keywords:** cognitive linguistics, prosody, coordination, social interaction, distributed cognition, ecological psychology, enactivism, distributed language

## Abstract

Using radical embodied cognitive science, the paper offers the hypothesis that language is symbiotic: its agent-environment dynamics arise as linguistic embodiment is managed under verbal constraints. As a result, co-action grants human agents the ability to use a unique form of phenomenal experience. In defense of the hypothesis, I stress how linguistic embodiment *enacts* thinking: accordingly, I present auditory and acoustic evidence from 750 ms of mother-daughter talk, first, in fine detail and, then, in narrative mode. As the parties attune, they use a dynamic field to co-embody speech with experience of *wordings*. The latter arise in making and tracking phonetic gestures that, crucially, mesh use of artifice, cultural products and impersonal experience. As observers, living human beings gain dispositions to display and use social subjectivity. Far from using brains to “process” verbal content, linguistic symbiosis grants access to diachronic resources. On this distributed-ecological view, language can thus be redefined as: “activity in which wordings play a part.”

“The important issue is not where cognitive processing begins and ends.”

(Vallée-Tourangeau and Vallée-Tourangeau, 2014).

## Introduction

Since it is beyond debate that living systems depend on metabolism, it can seem trivially true that cognitive activity draws on embodiment. To block any such view, the paper turns to *how* metabolism functions as “cognition emerges in ecological space and ecological time from the interactions of brain activity, motor actions, and artifacts” (Vallée-Tourangeau and Vallée-Tourangeau, 2014). In examining coordination in a multi-scalar ecology, it pursues Chemero's (2009) thesis that agent-environment dynamics ground all cognitive activity. In language, representation is replaced by emphasis on how persons concert activity or, simply, come to act as observers^1^. The resulting skills underpin the paper's thesis: language is activity based on symbiotic control of bodily movements that are perceived as “wordings.” Given phenomenal experience of iterated patterns, understanding connects the subjective to the impersonal or, alternatively, linguistic embodiment falls under partial control of a community's verbal constraints. Humans thus live in social meshworks of families, groups, communities, and even nations—each with characteristic ways of using linguistic embodiment.

While all embrained species interlace action and perception, in human groups, some of the time, and to some extent, people use “self-directed” “representational acts,” mimetic forms of activity that emerged millions of years before language (Donald, 1991). They arise under the control of one or more persons and contribute much to a community's forms of life. Ways of embodying mimetic performance appear in knapping flint, kicking a ball around, dancing, or taking part in talk. In each kind of activity, the results connect a human lineage with histories of individuals, relationships and ways of exploiting cultural complexity. Crucially, they link local and situated events to the products of a group's history. Unlike other primates, humans use extended cognitive systems. People draw on the past to alter later behavior: lived experience is enriched by using artifice and language to connect up the scales of time. Both distributed (Hutchins, 1995, 2014) and systemic (Cowley and Vallée-Tourangeau, 2013) approaches stress the multi-scalar nature of human cognition.

Culture enables people to tackle tasks by using structures that criss-cross temporal dimensions. A classic case is that of equipment that is designed to create images from deep-space (Giere, 2004). Human-technology aggregates enable the Hubble, for example, to connect the slow scales of physical evolution with the rapidity of light and mid-scales of embodiment and observation. In Giere's terms, *distributed systems* link artifacts, measuring, and the doings of human individuals. In spite of supra-individual complexity, the Hubble was made by and for observers. This is necessary to distributed systems: they depend on persons who use resources, including languages, to interpret what is perceived. Observers connect embodied measures with verbal skills to animate systems whose temporal scope reaches beyond lived experience. In what follows, this perspective is applied to human language-derived skills. Bodily synergies enable people to track and construe vocal movements: however, linguistic embodiment is also run through with affect. As people cooperate, compete, and otherwise coordinate, they set off nonce events that will be called *wordings*^2^. These phenomena arise as speech gestures constrain other aspects of linguistic embodiment. Crucially, they shape a richer phenomenal field: wordings co-occur with visible behavior and resonances as the vocal folds modulate the flow of air through changing vocal tracts.

People link linguistic embodiment with verbal and discursive patterns as they animate distributed systems. They do so both unthinkingly and as actors. While reiterating speech patterns, context can be used to attribute properties to so-called “words.” Eschewing appeal to representation or content, I thus liken the concept of language to the concept of mind. As proposed by Ryle (1949), I explain *belief* in mind and language without appealing to linguistic or mental systems “in the head.” Rather than posit dependence on neural dispositions, however, I stress the meshing of living embodiment with phenomenal experience. As a result it is possible to hypothesize that language is symbiotic: the phenomenal field is influenced, in part, by how people make and track phonetic gestures. By tracing the verbal to the phenomenal the paper rejects the code-metaphor^3^. or, in other terms, the view that “inner” systems process, generate or produce linguistic forms. Rather, embodiment links phenomenal experience to verbal patterns as, during ontogenesis, humans become actor-observers. In so doing, speaking and cooperating come under a degree of collective control. People gain skills in using a multi-scalar linguistic resource that allows embodiment to evoke impersonal products as people manage later events (cf. Hollan et al., 2000).

Since language is distributed across space and time, much can be learned from examining whole-body expression. To naturalize linguistic experience, one can thus begin with how bodily dynamics take on a verbal aspect. For, on this view, language arises as people coordinate and interpret events by linking movement with experience (Cowley, 2011b); the verbal is secondary and derived. In distributed terms, human cognition links interactivity (Cowley and Vallée-Tourangeau, 2013; Steffensen, 2013) with the normative sense-saturated flow of experience. Just as people learn to identify objects, events and situations, they learn to perceive reiterating phenomenal patterns, or wordings. As unique events, their sense results from experience with how a community uses phonetic gestures—events are heard against types that shape belief in words and languages. Humans become observers by learning to hear and articulate wordings. Once these are used to inform coordination, linguistic embodiment can shape activity around cultural goals and tasks. As a result, language is necessarily symbiotic. First, as emphasized below, linguistic embodiment is affective experience or a flow of direct meaning making. Second, its phenomenal aspect links phonetic gestures to wordings that allow descriptions of what linguists usually call language. Given a strange duality, language extends experience as people manage actions that are described by, but do not reduce to, the working of linguistic form and semantic content. The duality of wordings and coordinated action allows human cognition to reach far beyond the body. This is because, in concerting across time, people mesh verbal patterns with skillful action. Language has a multi-scalar heterogeneity akin to that of music, pottery or scientific practice. Humans exploit the scales of time as people who co-opt and transform material and biological structures. As a result, a global meshwork constrains activity in a staggeringly complex social world.

### Brief overview

My hypothesis is that linguistic embodiment and verbal constraints are symbiotic. Taking a distributed-ecological view, emphasis falls on, not linguistic forms (or content), but how cognitive resources are put to use in managing temporal experience. First, language is traced to the rapid or pico-scale dynamics that dominate linguistic embodiment: as shown below, it enacts measurable and observable bodily events. Second, parties are shown to use phonetic gestures, phenomenal experience and, given unending repetition, lay and linguistic *concepts* of language (*qua* verbal pattern). Further, while science cannot rest on faith in words, as argued by Sellars, Ryle, and Dennett (among others), just such beliefs underlie the social order and, thus, our accounts of human action. In tracing language to a symbolic-dynamical symbiosis, much can be shown to draw on co-embodiment. Accordingly, the core of the paper offers detailed description of events during 750 ms of dialog or languaging activity. Exploiting a pico-scale, mother and daughter coordinate by using voice dynamics that contribute to slower phonetic gestures (transcribed as [a:bεne]). These dynamics connect phenomenal experience of a wording with innumerable accounts of a verbal pattern or second-order construct (that can be written “ah bene”). The relevant praxis evokes a history of non-local events that gives each party her own understanding of what occurs. My hypothesis is thus defended by detailed description of how human observers use the symbiotic nature of language. This clarifies what “goes on” (at least roughly)–human actors make it happen. As languaging beings, we use beliefs about language (and languages) based on concerting actions and talk as part of living within the many scales of time (Madsen and Cowley, 2014). In this way, people gain subjective experience of temporality that enables culturally defined time to be used in action and perception. Human cognition is fundamentally diachronic.

## Language and the concept of language

“Language is first and foremost a temporal process whose dynamics and effects result from activity by two or more contextually situated individuals” (Fusaroli and Tylén, 2014, p. 1). In viewing it as a temporal process, language is allowed to permeate the scales of experience that bind people into a living meshwork that connects verbal patterns, social resources (e.g., money) and acting in ways that change the natural world. Much depends on cooperation between people who re-enact culture by using, for example, talk, texts, and output from language-machines. When relying on digital systems, human interactivity links multi-scalar dynamics to Shannon information. Importantly, computing depends on probabilities and, as Taylor (2012) shows so clearly, human talk also relies on statistical patterns. For Taylor, this confirms that form-meaning mappings use a mental lexicon. Denying the existence of a mental lexicon, the paper presents language as symbiotic. While disembodied *concepts* sustain intuitions and have enormous pragmatic value, their basis lies in, not individual minds, but using linguistic co-embodiment to grasp and sustain wordings.

Reified cultural templates (“languages”) dominated 20th century linguistics. This was because, just as people believe in tables and trees, they believe that wordings correspond to abstract objects (“words”). While having immense social and practical value, any such approach relies on lay views of language, intuitions or, as Wittgenstein (1957) prefers, “agreement in judgments.” In fact, like all folk beliefs, such views rely on reports of multi-scalar human coordination. In arguing against reducing language to a verbal aspect, I stress the use made of embodiment. As with other human activity, language exploits sense-saturated coordination: like colors or numbers, verbal patterns index highly socialized lived experience. While phonetic gestures lack the constancy of digits, like both these and color display, they use a history of synergies based on interpersonal coordination. On the view presented here it is precisely its amenability to description as both embodied and phenomenal that renders language possible. This is because, using appearances, grammatical, and statistical aspects self-sustain as living persons pass away over historical time. Phonetic gestures shape events that are lived as lexico-grammatical, pragmatic and probabilistic. Like number, language thus functions at a population level—in ways that change over historical time. However, in focusing on how phonetic gestures connect verbal constraints to linguistic embodiment, I stress how language lives through people or, indeed, how verbal patterns self-propagate through human coordination. This appears in a scale where people draw on cultural products to co-construct situations and lived experience. Pursuing this, I show how social, moral and linguistic products constrain movements by living beings or, in the terms of the paper, linguistic embodiment.

A naturalized linguistics begins with acoustic and kinetic measures—not reports about wordings (or “words”). Rather than favor “signs” over “substance,” language is traced to movement. Tracing the said to phonetic gesture, the paper presents 750 ms of talk during which a person utters [a:bεne]^4^. Analysis presents detail that shows why dialogical events are irreducible to intentions, phonetic gestures or experience of *a priori* types. While the intention is plain and [a:bεne] describes gestures made, the speech is symbiotic: events arise as verbal pattern constrains linguistic embodiment. Acting in a dynamic field, contributing to the flow of talk changes the layout of affordances. An act of utterance is joint activity which evokes what can be called linguistic “symbols.” In so saying, I echo Howard Pattee (see Pattee and Rąsczaszek-Leonardi, 2012), who, as a micro-physicist, traced language, computation and DNA to dynamics constrained by “symbols” qua self-organized measuring systems. On this view linguistic embodiment co-occurs with self-set control parameters. Yet, there is also a contrast between language and computation/DNA. Whereas computers and cells are self-managing (viz. they use phylogeny/metabolism and physical laws/human programming), language—and its symbols—depend on living human beings. Linguistic embodiment, coordinated action, speech, and hearing, is bodily movement that connects up central nervous systems. Skills in linguistic action arise as people couple control of airstream mechanisms, vocal folds, and articulatory tracts with phonetic gesture. Individuals act to connect metabolism with wordings and changing perceptions. As a result, skills in coordinating linguistic embodiment become enmeshed with what is learned from speech—people language by linking subjective experience with a grasp of the impersonal.

All embrained species exploit what Alain Berthoz (2012) calls perçaction. Action meshes with perception as, inseparably, people actively perceive the world. In humans, however, perçaction is transformed by language. Once utterance-acts are heard as reiterating patterns (as utterance-types) people mesh perçaction with experience of phonetic gestures (or wordings). They perceive objects and hear what the folk call “words.” The skills are learned; babies make sense of coordinated activity long before they make or track phonetic gestures. They come to use vocalizations to manage behavior and, in behaving, manage caregivers; they use rudimentary observing to manage how parties act, move and vocalize. By the second year of life, children co-construct “lived situations” and negotiate ways of “going on.” In parallel to Berthoz, wordings open up *observaction*^5^. Talk-in-interaction exploits utterance-acts that prompt multiple interpretations. In what follows, I focus falls on, not what such acts achieve, but their embodiment. I show how people use local control parameters (Pattee's symbols) and, without knowing what they are doing, evoke the linguistic, moral, and institutional resources of a community. By so doing, metabolism drives language. Embodiment connects the scales of time as people attune action with perception in activities that depend on coordinating finely regulated vocal and non-vocal expression:

- As perçaction language is sensorimotor activity that draws on/gives rise to rich phonetic memory (and its imagistic equivalents).
- As observaction, language is sensorimotor activity that draws on/gives rise to phonetic gestures (and their visible equivalents).

Phonetic gesture no more reduces to phonetic memory than rich memories of voice-speech-and-action suffice to explain verbal pattern. An utterance act like [a:bεne] influences social behavior whose dynamics *also* invite phenomenal experience (that is amenable to verbal description). Indeed, emphasis on event-experience symbiosis parallels Darwin's (1998) observation that language-expression is part-natural and part-artificial; in terms of the *Descent of Man* (Darwin, 1989), the ability to moderate natural sounds co-functions with mimetic abilities or, in his terms, imitation. Playing down both linguistic form and its derived artifacts (especially, texts and text-like “systems”), I pursue the Darwinian intuition by tracing verbal pattern to how phenomenal experience uses linguistic embodiment.

Instead of reducing [a:bεne] to linguistic forms, its acoustic and audible features can be attributed to the linguistic embodiment of Italians^6^. Indeed, those familiar with the “bel paese” will find themselves using this pattern of phonetic gestures in acts of utterance^7^. From a distributed perspective, the gestures that constitute [a:bεne] enact linguistic flow, a form of perçaction that permeates Italian ways of life. While [a:bεne] can be said unthinkingly, its uttering can also invite observation, construal, and interpretation. In the conversation described, it serves, in the main, as part of family events. By contrast, this paper subjects the same event to analysis. This is possible because [a:bεne] is symbiotic. It is at once:

- Rich phonetic (and visible) activity that is integrated in a family conversation.
- The issuance of an actor-observer (a wording) that can elicit observer-actor response.

Because an utterance act is symbiotic, [a:bεne] connects scales of human action. It can shape affective flow, enact a relationship and reflect on a person's family roles. This is possible because, like airborne synapses (Steffensen, 2013), its dynamics continuously enable and constrain how parties feel and act. Potential meanings—and a wording—trigger and result in a flow of phonetic gestures. The projecting, speaking/listening and gesturing of [a:bεne] is *direct* meaning making (see Cowley, in preparation). On a distributed view, [a:bεne] exemplifies sense saturated coordination. In making and responding to an utterance act, a mother and daughter are less concerned with construals (or “form”) than the richness of coordinated whole-body expression. At this instant, phenomenal experience frames events: far from inviting interpretation, the wording triggers subjective anticipation. It is not to be described by non-local meaning but, rather, by the particular sense it has for each party. While further discussed below (in Section How [a:bεne] Functions), far from reducing to truth-conditional acts (see Oaksford and Chater, 1991), human action draws on essentially subjective probability estimations (Madsen, 2014). Bayes' theorem is a normative description whose probabilistic estimations describe, not a brain's workings, but how a person anticipates. Linguistic experience—and interpretation—thus builds on concerted embodiment. Since subjectivity is inherently social, it has a central role in cognition that extends beyond the body.

Subjectivity uses embodiment in all forms of perçaction (e.g., looking). Cognitive events such as those based on saccading or looking depend on time-scales at and below awareness^8^. In language too, action uses concerted looking as affect links utterance acts while setting off resonances and damping-effects. Coordination links looking and talking as people establish consensual domains (Maturana, 1980) or, alternatively, develop shared discursive and other practices. In short, during coordinated action, people also gain the skills of human observers. For example, they may come to see the point of actions or, indeed, to grasp the sense of various ways of displaying intentions and attitudes (and the microsocial order). As a result, they share beliefs about language: they *picture* forms and meanings as part of the world. Concerting bodies while attending to wordings thus links perçaction with forms (and *concepts*). Just as with monetary values or musical offerings, people draw on probabilistic information. In an attested, mundane example, a suspect can reasonably refuse to give a policeman information when he has admitted his guilt. In these circumstances, not naming confederates is licensed by the normative order (see Edwards and Potter, 2005). Since this is legitimate and intelligent, the management of social roles must be deemed “cognitive.” An observer draws on circumstances to decide what he need not say. Much the same applies to the unsaid. Since language is symbiotic, observers may focus on acting like a policeman, speaking English or (not) sounding Liverpudlian. The language flow is cognitive in that it affects the unfolding of lived experience. Thus, as with refusing to give information, a person may speak in order to sound educated: the cognitive influences social judgment. Indeed, since practices link human embodiment with the verbal, the artificial becomes social. As Hutchins (1995, 2014) shows, in settings like the cockpit, cognitive events are dominated by slow processes. In Wittgenstein's terms, the cockpit links language games with forms of life as pilots perceive *aspects* of the world. While cognition is enabled by embrained bodies, cultural resources prompt meaning making as an observer individuates what is important. By linking natural, social and material resources, the products of past events change later activity. A 4 million year history of self-directed representational acts (Donald, 1991) influences evaluation and learning as people self-improve and cooperate. Human motivations induce practicing and, thus, people gain fine control over vocalizations—the grounding of musical, mathematical and linguistic extensions to embodiment. The lived experience of language thus meshes with beliefs and conceptual tools that arise in a community's praxis.

## Linguistic embodiment

Linguists link lay views with Saussure's authority to build linguistics on phenomenal experience of how phonetic gesture can be transcribed. Invoking abstract objects (e.g., words, generative grammar, conceptualization, or I-language), they invoke, not acts of speaking-while-hearing, but abstract types (e.g., utterances, sentences, constructions, usage-patterns). Emphasis on words and rules thus divides the linguistic from the non-linguistic. By fiat, sense-saturated coordination ceases to be language; linguistic embodiment ceases to be linguistic embodiment. If acknowledged, bodily dynamics are ascribed to modalities or paralinguistic and prosodic systems. Conversely, on a distributed perspective, language *is* sense saturated coordination whose neuro-social constraints sustain observing—activity in which wordings play a part (Cowley, 2011b). It emerges from the synergies and movements of linguistic embodiment that shape a flow of activity during which both macro and micro constraints affects what people do. People may speak and hear, for example, as part of a family: talk coordinates action (and vice versa). Linguistic embodiment has a role in constituting phenomenal experience that uses neural, microsocial, and cultural constraints. It enacts social activity and, paradigmatically, conversation. The claim is readily defended. First, talk is of pivotal concern to most people. Second, conversations ground skills that depend on language (e.g., flying planes, seduction, hunting). Third, talk is almost certainly the basis for the phylogenetic emergence of language—perçaction based on linking airstream mechanisms with control of the articulators. Not only is this a Darwinian view, but it allows cooperation and cognition to derive from coordinated movement. As with dance, music, and sport, language uses cultural and bodily constraints to social effect. Although literate people picture language as it “can be separated from its material expression” (Thibault, 2011, p. 2), this dubious surgery strips it away from lived experience. By leaving aside how people use utterance-acts, language is excised from the ecology: it is forgotten that “thinking depends as much on the environment of the thinker as it does on his or her brain” (Wells, 2006, p. 2).

Let us consider how co-activity draws on a single uttering of what can be described as [a:bεne]. In offering a little detail about these 750 ms, I show two segments (see colons) exploit audible pico-scale lengthening. Whereas the initial “b” is striking, the long [ε] vowel is typical of the speaker^9^. For the speaker's mother, the latter is thus unlikely to be perceptually salient; further, letter-spacing hints at other pico-scale timing (thus, “a h” is slow). Moreover, while hundreds of measures could be reported, the transcription picks out acoustic correlates of pitch on the first and last measurable vowels [Cowley's (1994); interchange (IF) and enjoining (EF) frequency]. Finally, there is a marked fall on the prominent syllable. (All measures are given in Hz).

**Figure:**
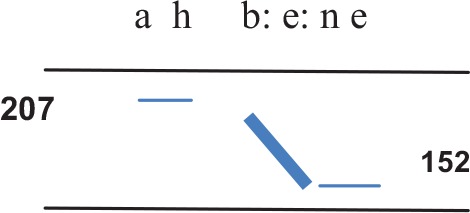


As part of mother-daughter “thinking,” the utterance-act binds what precedes with what is likely to follow. Speaking [a:bεne] is a “striking example of human inventiveness” (Cowley, 1994) where discursive practice uses human musicality. While the initial pitch (207 Hz) is near the daughter's norm (her mean IF is 215 Hz) and the prominent falling tone unexceptional (compare unmarked “oh good”), the act is striking. The lengthening of [b:] is an emblem of status that prefigures a “decisive” fall of half an octave (on *bene*). Indeed, this drops from about the daughter's mean IF a full standard deviation below her norm (152 Hz)^10^. Importantly, her speech rate *matches* her mother's almost perfectly: while her mother's rate is 240 ms per syllable, the daughter's is 250 ms. And, as emphasized in the Section “How [a:bεne] Functions,” the “meaning” is also striking. Since thinking and social events are partly constituted by linguistic embodiment, details show more than sophisticated speech timing. Crucially, as phonetic gestures attune to her mother's voice, the voices create inter-individual patterns. Once these coordinated dynamics are noticed, one sees that, far from being paralinguistic, their musicality affects how the parties act, feel, and verbalize. Thus, [a:bεne] is co-constructed or, in another idiom, an other-oriented act (Linell, 2009). Human dialogicality neither reduces to conventional use of form/meaning nor to typologies of speech act. No “pure” linguistic or cognitive model can show how mother and daughter coordinate. In Levinson's (1995) terms, this is interactional thinking: the sense of [a:bεne] is enacted (i.e., not inferred from the context of “ah bene”).

Although amenable to separate analysis, the so-called modalities co-constitute speaking-while-listening or first-order languaging. Indeed, on the distributed-ecological view, interactivity shapes *experience* of talk. Its sense-saturated and normative aspects enable bodies to coordinate peoples' feelings, thinking, and acting. Crucially, the dynamic field of experience involves more than phonetic gesture. Even if one leaves aside visible behavior, people use rhythmically based pico-scale dynamics based on modulating the air stream mechanism while making phonetic gestures. Saussure's error lay in dividing form from substance or, without argument, unzipping breathing from vibration of the vocal folds and how a changing vocal tract constrains phonetic gestures. Linguistic embodiment exploits a speaker's whole body movements. Indeed, it is a scandal that phenomenal experience is often blithely assumed to confirm the “reality” of a language-system. In ignoring linguistic embodiment, a focus on “form” echoes Cartesian dualism and debates about “representation.” As a result, many 20th Century linguists ascribe language to the mind—echoing rationalist or empiricist debate. The radical nature of Chemero's view of embodiment is that agent-environment dynamics, not brains, become the basis of cognition. Language thus begins with pico-scale sensorimotor control that allows wordings (and inscriptions) to be derived from phonatory control, movement, and phonetic gesture. Indeed, it is a simple fact that people hear utterances as reiterations of the latter: given motor skills, the brain is trained through re-use^11^. Repetition of speech fragments in strategic social action attunes phenomenal experience to the movements that sustain and echo collective life. In lived experience, people thus draw on the past, invoke the future and exploit the impersonal. As with mother and daughter, meaning-making is direct and idiosyncratic. *Contra* Lyons (1977), language reduces to neither standardized, regularized nor decontextualized forms. While such models highlight the half-artificial, they overlook how bodies to move each other in a pico-scale. In fact, as with [a:bεne], much uses what Abercrombie (1967) calls voice dynamics, continuous phonetic fluctuations that modulate the said. Prosody is thus redefined as “aspects of an individual's speech explicable neither in relation to word-based forms into which the speech can be analyzed, nor as part of the invariant auditory coloring that identifies an individual speaker's voice” (Cowley, 1994, pp. 6–7). As linguistic embodiment, meaning spreads as people exploit pitch, loudness, pace and so on. Like a cultural artifact or brain, human musicality serves as a cognitive resource. People show exquisite sensitivity to voices as they co-operate, talk, and manage emotion. Since voices serve in action, the results shape joint procedures and, thus, social events. In everyday life, dynamics connect wordings with circumstances, history and what is manifestly heard. The symbiotic coordination of “language” derives from, not verbal patterns, but bodily achievements: it is activity in which wordings play a part.

### How [a:bεne] functions

Linguistic embodiment involves much more than phonetic gesture. In presenting a single “interact” (Linell, 2009), the case of [a:bεne] shows how phonetic gesture can be subordinated to a pico-scale flow. However, it is also important to sketch how coordinated thinking draws on the richness of lived experience (for more detailed description, see Cowley, 1998; Cowley, in preparation). In what follows, therefore, I place how the women act within a wider event trajector. In so doing, I use transcription to build a narrative gloss:

**Table:** 

M : Qu**e**sti so**n**o del **tuo or**to?	M. Are these from your garden?
A: **Oeu** me ne ha **da**to	A: Mmm, she gave me
un **po’**	a few did
la **Ro**sa #	Rosa #
ce ne ha **da**to	she gave a
una bor**sas**sa	ginormous bag to
la Pal^**mi**ra^	Palmira
**M. Ah bene**	**M. Oh good**

Briefly, having asked if the peas they are eating come from their garden, the daughter soon realizes that this is a mistake. Her mother begins to launch into a lament—they are not and, what's more, she only got a few while, worse still, Palmira was given a “ginourmous” bag of peas^12^. As the daughter says “ah bene,” she attempts to control her mother. Thus, in terms of content-pattern OH GOOD is anomalous: giving a positive spin to events (“good that she gave you a bag”), the daughter seeks to deflect a train of thought. At the same time [a:bεne] enacts how she feels—affect permeates gesture, wordings, and facial activity. Crucially, the act thus depends on pico-scale voice dynamics, the metabolic underpinning of language. It is in this sense that the importance of the phonetic detail lies in how the daughters' utterance-acts come to be suffused by her mother's co-presence. Broadly, the utterance-act *is* the thinking or, alternatively, speech enacts meaning.

Many linguists focus on how a person can perform the same acts over and over again. In prioritizing what Colunga and Smith (2008) term the *problem of stability*, they reduce prosody to patterns and, overlooking voice dynamics, emphasize discursive, and intra-utterance regularities that are said to generate rhythmic and tonal patterns (e.g., how tone groups map onto prominences and patterns of pitch, duration, and loudness associated with marked syllables). While said to be “communicative” (whatever that means), models of prosodic systems are powerless in clarifying function. This is because, in focusing on the recurrent, they make pico-scale voice dynamics “paralingistic.” The models disembody language by separating it from experience. On the distributed-ecological view, by contrast, the dynamics of language flow shape how parties “decide what to say.” As shown in fine-grained analysis, extensive use is made of rapid bodily attunement, improvisation, and lived relationships. Indeed, this enacts most of what we call emotion, attitude and how people vary the deliberation (and inhibition) of social life. Of course, as an embrained species, humans use learning and repetition; yet, as observers, individuals also use particularities. Language exploits interactivity or, for Colunga and Smith, dynamics permitting us “to smartly do novel things that integrate the stabilities of past experience with the idiosyncrasies of the moment” (2008, p. 175).

Metabolism reasserts itself as the talk continues through the 750 ms during which the mother finds a way of going on. In so doing, she pointedly pays no attention to the phonetic gesturing. Far from speaking up on the positive, she speaks as if her daughter had *said* nothing. Indeed, using the voice dynamics, she redoubles her complaint. Far from relying on interpretation, this is affective vocal expression: the parties co-enact flexible, adaptive behavior that alters neural processes, sets up priors, and shapes subjective experience. Not only do they know what Everett (2012) calls “the joy of language” but their speaking and moving is thick with sense. Phonetic gestures intermesh as people take each other's measure–sensing how they are assessed. Interactivity affects feeling, thinking and acting in scales that are more rapid than phonetic gestures and audible shifts in tones of voice. While a micro-scale highlights what we articulate (syllables, tone-units/phrases, and utterances), much depends on people whose resonating voice dynamics set off sound patterns with variable probabilities^13^. Thus, attention shifts to functions that characteristically occur in 50–200 ms range: in this pico-scale interpersonal synergies are ubiquitous. Living language is grounded in, not wordings, but bodily movement. Of course, linguistic embodiment is no more than a necessary part of language—at times people choose to say, write and construe things with much more deliberation. That too must be considered.

## The elephant in the room

There is nothing exceptional about tracing human activity to *how* cognition emerges in ecological space and ecological time. Not only is this also true for sport and dance but, not surprisingly, it is also applicable to problem solving. As shown in experimental work, people use interactivity, sense-saturated coordination, that mediates embrained bodies, motor actions and artifacts (Ball and Litchfield, 2013; Vallée-Tourangeau and Vallée-Tourangeau, 2014). In talk, much depends on pico-scale dynamics (Cowley, 1994, 2010; Thibault, 2011; Steffensen, 2013; Cowley, in preparation). By implication, linguistic embodiment enables living subjects and communities to use musicality as language arises *beyond* the body. Next, therefore, I turn from lived dynamics to the non-metabolic. The point is that embodied musicality suffuses what are heard as physically-based patterns like “*ah bene.*” Pico-scale events shade phonetic gestures as utterance-acts become audible as utterances *of* something. To repeat the mantra, language is activity in which wordings play a part. However, one must be careful: wordings emerge as those familiar with, say, Italian forms of life hear phonetic gestures. While reported as linguistic types, these function, not as “forms,” but nonce events. Like numbers or colors, phonetic gestures are resources used in action. Indeed, linguistic embodiment gains its power from managing how one languages in ways that evoke a community's speech patterns. In distributed terms, second-order constructs (i.e., lexical, semantic, phonological, morphological, syntactic, pragmatic, and stylistic patterns) constrain what people do, feel and, thus, think. Further, much of the time, of course, people act “mindlessly”: as a result of a life-history, they adopt beliefs in the reality and power of wordings. In literate communities, these become associated with inscriptional forms such as “ah bene.”

As activity in which wordings play a part, language becomes insinuated into almost all areas of human life. In more formal kinds of talk, worship, text-messaging, for example, the verbal dominates. Indeed, without skill in perceiving wordings, there can be no human observers—and no language. In ontogenesis, skill in perceiving phonetic gestures as wordings arises from experience of interacts (Linell, 2009). On a distributed-ecological view, the perceptual skill arises in zooming out of a full-bodied situation. Thus, while utterance-acts link metabolism to local practices, they also come to be heard in a particular sense. While these can be ascribed to intentions, this is a second-order model. In the case of [a:bεne], it is a fact that, for Italians, the act evokes hearings of “ah bene.” While further discussed below, I stress only that prompts and probes exploit meaning potentials (Linell, 2009). The daughter finds herself moved to stop her mother: her polyphonic [a:bεne] links Italian ways of managing conflict with a mother-daughter relationship; it enacts her concerns, her perception of her mother and, indeed, a wish to be “positive.” The holistic nature of [a:bεne] links neural synergies with habit as phonetic gesture binds various time-scales into a lived situation. As a chunk of behavior, its verbal aspect resembles a *real-pattern* (Dennett, 1991; for development, see Ross, 2000). Functionally, the utterance-act triggers pattern recognition that bears the hallmark of reward-based learning. Dennett (1991) compares this with how von Neumann machines use zip files; instead of applying a value to every bit of information, programs use compression. By analogy, hearing [a:bεne] calls up senses that *may* be valid. Voice dynamics constrain experience and thus prompt anticipation. In the flow of talk, wordings—the phenomenal experience—index ways of proceeding. Just as a computer needs users or a cell an environment, language demands an observer. Like a zip-file, [a:bεne] evokes compressed information if, and only if, a person finds a *perspective* from which the pattern can be used.

Wordings exist as they are perceived. As phenomenal experience, they carry a particular sense which has little in common with either the meanings or forms that linguists ascribe to verbal patterns (second-order constructs). Yet, as phenomenal experience, a wording calls up historically derived affordances as embodiment and circumstances prompt ways of going on. People use partial control over phenomenal experience to concert their movements. As in music or dance, they jointly manage how the rapid scale of linguistic embodiment resonates with historical events as, in pico-scales, neurophysiology enacts coordinated activity. The symbiotic nature of language thus connects two kinds of reward-based learning. On the one hand, like rats and wolves, people learn both individually and socially: they use exposure, probability judgments and rewards. On the other, people also use wordings in learning by observing. They notice, and prompt each other to notice, aspects of the world. They use anomalies and turns of phrase; they pick up on attitudes, intentions and hidden parts of the environment (e.g., urgency, potential for use). Observation-based learning, it seems, is specifically human. It enables the use of abstract qualities (e.g., bene, red, strong) to connect the more intuitive to the more deliberate in, for example, telling a joke or proposing another round of beer. Phenomenal experience thus offers valuable ways of gauging how to act in the circumstances. Although dealing with [a:bεne] is largely a matter of co-embodiment, the example represents relatively automatic talk. On many occasions, human intercourse depends on much closer attention to wordings. In stories, for example, wordings dominate narration. In other settings, they are even more basic—for example, they sustain writing-systems.

Wordings can be used in literal, poetic and hypothetical ways; in contrast to dealing with [a:bεne], people can choose to rely on the “words that are actually spoken.” As argued elsewhere, much can be gained by taking a distance from the flow of talk by focusing on phonetic gestures and seeking to repress the unsaid. In so doing, people learn to take a language stance (Cowley, 2011a). While talk fluctuates between greater reliance on automaticity (as in the 750 ms of [a:bεne]) and more careful use of a language stance, there are also intermediate modes of acting and attending. People can and do shift emphasis between the said, what they say and, indeed, the unuttered (“silent thoughts”). In its impersonal aspect, language opens up *other people's* experience (see Cowley, 2014). For example, if I allude to Endel Tulving, informed readers may evoke mental time-travel: in its verbal aspect, the inscription *Endel Tulving* compresses ways of going on. By opening up an impersonal past (using “priming”), the reader's embodiment anticipates what is likely to follow. Skills connect automated perception with how observers construe circumstances. Since wordings draw on (or resemble) real patterns, particulars can be perceived as types or “sames.” While real-patterns evoke reports of hearing (and seeing inscriptions), they are equally likely to affect how mother and daughter concert their speech. As they do so, people enact and display experientially-based modes of practical understanding^14^.

People use fine phonetic information to perceive, not an utterance act's particulars, but “salient” details that serve to anticipate circumstances. In making rapid judgments about [a:bεne], both parties use compressed information associated with statistical experience. Far from using all available “information,” people note sensitivity to unmet expectations (or expected standards). As a result, combining real-patterns with voice dynamics (not to mention movements of face and gesture) contrasts with deliberate use of historically derived patterns. Whereas prosody is usually managed by ear, observers can shift attention between the said, the words that actually spoken, and how they hope to sound. Wordings offer a degree of individual control: one can even speak impersonally. While consistent with folk wisdom, psychology, linguistics, and cognitive science are guilty of overlooking such phenomena. Avoiding dynamics, they trace phenomenal experience to skills in monitoring the said, projecting what is likely to be appropriate, and choosing how to “come over.” In turning to the verbal aspect of language, as opposed to how [a:bεne] is co-embodied, groups can be said to draw on a “systemic” meaning potential (Halliday, 1985). Since this echoes the slow scales of history, to the extent that a subject grasps this potential, he or she can use wordings to recalibrate acting, thinking, feeling other forms of self-display.

Once language is recognized as symbiotic, one begins to rethink the verbal. First, since perceived wordings can be repeated and analyzed as parts and procedures, phonetic gestures allow both skilled hearing and strategic use of utterance-acts. From a distributed perspective, these shape perçaction—language is skilled action. By implication, mechanisms beyond the brain function as people use phenomenal experience to coordinate activity. Language thus sustains the people of a social meshwork: the verbal aspect of talk uses a time scale where people enact organized social practices (Enfield, 2011). However, [a:bεne] also arises as a mother's voice moves her daughter to use cultural resources. Though hearing the same phonetic gestures, each party reacts differently. Linguistic symbiosis allows social factors to work through people whose interactions shape circumstances, relationships and the Italian life. Thus, while connotational meaning is precise, both women draw on experience of tens of thousands of similar cases: these constitute a fuzzy denotational meaning (OH GOOD). At this moment, however, this matters little. Events link the emotional interplay to circumstances and the mother ignores her daughter's “positive” move.

### Verbal patterns are partly shared

Since people have similar experiences, verbal patterns come to be partly shared. Further, given rich phonetic (and visible) dynamics, circumstances influence how people assess and manage each other. In construing a single act of utterance, the women draw on how countless hearings of phonetic gestures enrich experience of both Italian forms of life and their relationships. In so doing, they attend to what can be written as “ah bene.” This verbal pattern can be described at the population or corpus level; even here, however, it is not purely verbal in that, among other things, it evokes attitudes and probabilities. In Italy, the pattern's penumbra thus sets off relatively predictable effects. When one experiences a wording that can be rendered as [a:bεne], perception connects up with scales of time and thus cognition beyond the body. Far from using a shared lexicon, mental or social, the parties rely on making and tracking phonetic gestures. Given the symbiotic nature of human language, no more understanding is required. Rather than ascribe a causal function to verbal patterns, they are second-order constraints on lived experience. Like numbers or colors, wordings link indices of past events to circumstances or, in Maturana's terns, trigger connotations. Far from using tokens “in the head,” as (or like) real-patterns, wordings trigger events over which a person exerts some control. During talk, parties manage and inhibit their promptings, in part, by “choosing” what to say. People rely on activity in which wordings play a part to grasp and alter what they and others perceive, mean and say. Familiar ways of speaking/acting and associated probabilities offer some control over actions. The perceived—wordings, colored objects or analog/digital “representations”– need do no more than evoke an iterable pattern. Skills in conjuring up the audible or visible aspect of language thus ground what Wittgenstein (1980) came to call “certainty.” Human modes of life, and living bodies, enable one to make and accept utterance-acts such as: “My name is NN” or “I have never been to Bulgaria.” Indeed, people can even play philosophical games by making explicit judgments of whether or not it is appropriate to say, “That is a tree.” Crucially, such claims become transparent only to the perspective of an informed observer: in themselves, they are trivial. It may be *true* that a philosopher is pointing at a tree and saying what it is and yet, at the same time, appear quite pointless to act this way. If *that* is to be explained, human forms of life need to be traced back to interactivity: one must show how people become observers who, to an extent, share a perspective on phenomenal experience. By hypothesis, this is possible because of symbiosis between linguistic embodiment and the verbal. Utterance-acts evoke wordings that, via phonetic gestures, allow people to connect their co-embodiment with impersonal and shifting verbal patterns.

Language is typically approached from an observer's perspective. In the terms used here, a person takes a language stance by attending to behavior, or products of behavior, that derive from phonetic gestures. From this stance, language can be discussed, re-described, formalized and, in slow time scales, transformed. In history, utterance-acts change in parallel with conceptual evolution. Slow events constrain how perçaction shapes individual experience: thus, while non-linguistic “thinking” appears in many embrained species (see Bermúdez, 2003), humans use new kinds of thought. Drawing on phonetic gesture, children hear wordings and, eventually, develop skills based on the language stance. This enables preferences to be connected with beliefs as children learn how, in various settings, things are done. As a result, they can develop ways of competing, coordinating and cooperating. They may discover, for example, that the same phonetic gestures allow a shirt, hair, or wine to be called “red.” In so doing, they gain access to the concept's impersonal aspect (“redness”); however, like the taste of wine, the smell of hair and the look of a shirt, this is also subjective. Whilst the phenomenal moors certainty, social encounters permit a slow accumulation of conceptual understanding. Utterance-acts call up experience based estimations of how wordings will be heard or projected meaning-potential. As a person orients to others, they approach wordings as observers. While influencing talk, they also elicit construals as, over time, each person gains a sense of semantics. In the philosopher's garden, people match judgments by connecting well-timed pointing to, for example, saying, “That is a tree.” Of course, philosophers often erroneously seek to “explain” this referential relation. Yet, on the distributed view, though languaging is subjective or connotational (but not private), communities also exploit collective and denotational meaning. As shown by the mother and daughter, linguistic embodiment arises as concerted movements and voice dynamics shape a flow of social events. In the case described, while giving little attention to the words actually spoken (“ah bene”), the parties re-enact their relationship–as only Italians can. It is by virtue of the symbiotic nature of language that the parties grasp value-labels (e.g., BENE/GOOD) that serve to sustain a cultural lineage and, in so doing, a bundle of social practices.

## Human cognition and the scales of time

The paper shows that, in studying cognition, one can ask *how* embodiment functions. Applied to language, talk is seen as intrinsic to a history of interactions that connect sensorimotor activity, brains, and forms of human artifice. As in the mother-daughter exchange, linguistic embodiment connects parties across the scales of time. In this sense, language is symbiotic or, simply, linguistic embodiment connects movement with experience of wordings. There are, at least, two reasons for which the claim is non-trivial. First, symbiosis permits coordination between and within individuals: as this occurs, the women relate to each other. Using their voices, they concert expression and, thus, evoke meaning potentials that observers associate with verbal patterns. This leads to the second point. An evolutionary history links phonetic gestures with phenomenal experience such that wordings connect subjective experience with an impersonal aspect. During talk, people engage with each other and, to varying extents, use language reflectively. By taking a language stance, they can contribute to (or inhibit) debate about verbal and conceptual patterns. Affect and whole-body dynamics thus link human vocalization with impersonal experience that grants access to species-specific resources. Given linguistic symbiosis, cultural products can be re-used at later times (Hollan et al., 2000). In contrast to other primates, members of *homo sapiens sapiens* link embodiment to artifice as they shape relationships, institutions, and the cultural ecology.

The polyphony of language not only grants access to language machines and texts but it makes individuals part of a cultural heritage. People use this to draw on compressed information that pertains to the world beyond the body. This arises because, like zip files, phonetic gestures facilitate mental time-travel by calling up both personal and impersonal experience. As Merlin Donald saw, the evolution of a cognitive-cultural network transformed human intelligence: it made it possible to create and deflate possible worlds Once verbal patterns are insinuated into embodiment, language can self-sustain in a collective or population domain. People live in language as they coordinate within a meshwork of bodies that link ecological space with ecological time. Children participate in distributed cognitive systems and, as Giere (2004) insists, they do so as human agents. By hypothesis, human cognition is transformed as linguistic symbiosis allows them to develop the skills of observers. Unlike other primates, the mother and daughter orient to [a:bεne] and, in the space of 750 ms, co-construct a situation that re-enacts their relationship. Polyphony enables them to act strategically and cooperatively. By hypothesis, they use compressed Shannon information that is phonetic (e.g., durational), verbal (e.g., based on usage and discourse practices), and conceptual (e.g., exploiting semantic attributions). Using phonetic gestures, wordings sustain the ways of speaking used in Italian communities. Humans thus use cultural resources to construct new kinds of temporal experience. It is because meaning making is ecosystemic that, for example, astronomers can explore the history of the universe. Further, as affective, interpreting beings, people link embodiment with wordings. Distributed systems enable living communities to build collective memories and specify possible futures. Brains are recalibrated as people use priors that sustain reasoning. Individuals become living subjects as embodiment connects people within a social meshwork.

Humans are strange. In most species that use social learning—rats, wolves, or elephants—collective intelligence centers on individuals. In humans, by contrast, much depends on an evolving cultural or impersonal domain. This is because, while based in embodiment, activity draws heavily on reports of how wordings contribute to experienced phenomena. People connect perçaction with skills based on using a language stance: they strategize, refine values, and develop social practices. For this reason, acknowledgement of linguistic symbiosis offers much to radical embodied cognitive science. Mental content is replaced by treating language as activity in which wordings play a part. People use embodied coordination together with phenomenal experience of wordings. They need, not neural representation, but dispositions that link neural resources to the world beyond the body. Human intelligence exploits diachronic agent-environment dynamics. Given linguistic symbiosis, perçaction enables human individuals to use wordings in observation. Individual lives can be regulated around impersonal resources. Not only do we conform to social practices, norms and beliefs but, crucially, artifice, and wordings enable individuals to self-configure. As Heidegger saw, *experience* of language makes humans distinct. As we exploit the accountable, meaning potentials arise—people come to believe in languages, minds, and mental content. Social life uses such beliefs, above all, to draw on past—mythical, lived, told and impersonal. Its imagined outcomes can thus be put to use in making futures. This allows experience to be recalibrated as when, for example, philosophers pursue enquiry by pointing at plants in a garden while uttering variations on “That is a tree.” Remarkably, language lays down markers for possible futures as people navigate ecological space and ecological time. Drawing on interactivity, history and wordings, each one of us becomes a living subject who, for a moment, exerts some control over who and what we become.

### Conflict of interest statement

The author declares that the research was conducted in the absence of any commercial or financial relationships that could be construed as a potential conflict of interest.

## Footnotes

^1^People do not just engage with the world: they also treat what they perceive and how they act as making sense and/or meaningful (e.g., Maturana and Varela, 1994).

^2^The paper builds on the Browman and Goldstein's (1992) articulatory phonology in that the verbal aspect of speech is traced to how people make and track phonetic gestures (based on timing of articulatory movements in a moving vocal tract). Not only is there massive support for the view (see Fowler, 2010) but it abolishes mental representation. More controversially, I treat this as compatible with Port's (2010) demonstration that people use rich phonetic memory –a single utterance-act evokes many phonetic exemplars. Given the ecological basis of such views, the paper's perspective is deemed distributed-ecological.

^3^Appeal to the code view is shorthand for reducing linguistic activity to the systematic use of linguistic forms (whether cashed out in behaviorist or cognitivist terms). Variations on the negative argument are set out in Harris (1981); Linell (2005); Love (2004), and Kravchenko (2007). Interactivism offers a related perspective that derives from the context of cognitive science (see Bickhard, 2009).

^4^This moment of first order languaging was chosen for two reasons: first, its striking inventiveness is relatively independent of what is said—a daughter shows exquisite skill in trying to head her mother off her conversational path. Second, for Cowley, the case exemplifies an utterance act that enacts meaning directly as prosody begets prosody.

^5^Didier Bottineau (personal communication) suggested this extension to Berthoz's (2012) work.

^6^Nigel Love (personal communication) argues that, somehow, Italian speakers share considerable knowledge about how verbal patterns (the second-order constructs of Italian) are used. Use of [a:bεne] is, he thinks, inseparable from whether the parties believe it consists in, say, one or two words. He and I differ on two parallel points: I claim that people are, at once, caught up in flow and, at times, use observaction; he emphasizes deliberate speech and interpretation. Accordingly, I emphasize that [a:bεne] is heard by tracking phonetic gesture as part of whole-body experience; he emphasizes that, for Italians, any instance of [a:bεne] evokes abstract units that pertain to a community's speech. For the same reason, Love makes no distinction between “wordings” (*qua* nonce phenomena) and verbal patterns (*qua* constructs that describe community practice).

^7^Bechtel (2008) offers a book length account of how the biology of cognition can be explored mechanistically. On a distributed perspective, the verbal aspect of language is a *mode of organization* that constrains the workings of bodily *parts* and the biocultural *procedures* that serve to insinuate language into a range of resources (i.e., by linking face-to-face interaction to both texts and various kinds of language-machines).

^8^Emphasis on the pico-scale or how vocal and other gestures are made is characteristic of the distributed view. Pioneered by Cowley (1994), the approach is increasingly influential (for example, Thibault, 2011; Uryu et al., 2014).

^9^While Lombard, the speaker usually uses a Northern version of standard Italian which is striking, in part, for marked use of geminate consonants: as a schoolteacher she often speaks in ways that contrast with the local dialect (where there are no geminates). Her unusual lengthening of the “b” on “bene” (as in central and Southern Italian speech) distances her from her mother's use of the dialect form “borsassa” (-assa is a widely used negatively charged suffix that denotes large size).

^10^Detail is offered in a paper (Cowley, in preparation) that uses this passage to show how linguistic embodiment (or first-order language) enacts *direct* meaning making. Details like those reported are ubiquitous in family conversations (see Cowley, 1994).

^11^As multiscalar activity, language unites many kinds of network. It is likely that, with skills in speaking, listening and otherwise drawing on linguistic resources, people “rewire” or sculpt their brains as suggested by Anderson's (2010) hypothesis of neural reuse.

^12^As noted above “borsassa” is a dialect form that gives negative connotations to an object of some size. In order to render something of this, I have translated it as “ginormous.”

^13^In many species, Hinde (1979) informs us, much depends on both the probabilities with which individuals do things and, at times, on how they are done. Though primate grooming is the classic case, birds also exploit both micro–and pico-scales–a clear case is that of how Alex the parrot taught himself to say “spool” (see Pepperberg, 2007).

^14^The Dennett-Ross view of real-patterns recalls Pattee's symbols in that these too are measures that constrain dynamics in observable ways. However, in generalizing from DNA and computation to linguistics, Pattee views linguistic symbols as brain internal. By contrast on the Ross-Dennett view, while voice dynamics are public, cultural real-patterns (including wordings) evolve in populations: they encompass language, traditions, music, money, etc.—not to mention associated procedures and institutions.
